# Development and validation of an online tool for assessment of health care providers’ management of suspected malaria in an area, where transmission has been interrupted

**DOI:** 10.1186/s12936-022-04308-1

**Published:** 2022-10-27

**Authors:** Hosein Azizi, Reza Majdzadeh, Ayat Ahmadi, Ahmad Raeisi, Maryam Nazemipour, Mohammad Ali Mansournia, Allan Schapira

**Affiliations:** 1 Department of Epidemiology and Biostatistics, School of Public Health, Tehran University of Medical Sciences, Tehran, Iran; 2 Research Center of Psychiatry and Behavioral Sciences, Tabriz University of Medical Sciences, Tabriz, Iran; 3 School of Health and Social Care, University of Essex, Colchester, UK; 4 School of Public Health, Knowledge Utilization Research Center, and Community Based Participatory Research Center, Tehran University of Medical Sciences, Tehran, Iran; 5 Knowledge Utilization Research Center, Tehran University of Medical Sciences, Tehran, Iran; 6 National Programme Manager for Malaria Elimination, Department of Parasitology and Mycology, School of Public Health, Tehran University of Medical Sciences, Tehran, Iran; 7 Bicol University College of Medicine, Legazpi City, Philippines

**Keywords:** Malaria elimination, Suspected malaria, Health workers, Simulated patient, Online tool, Validity

## Abstract

**Background:**

The alertness and practice of health care providers (HCPs) in the correct management of suspected malaria (CMSM) (vigilance) is a central component of malaria surveillance following elimination, and it must be established before malaria elimination certification can be granted. This study was designed to develop and validate a rapid tool, Simulated Malaria Online Tool (SMOT), to evaluate HCPs’ practice in relation to the CMSM.

**Methods:**

The study was conducted in East Azerbaijan Province, Islamic Republic of Iran, where no malaria transmission has been reported since 2005. An online tool presenting a suspected malaria case for detection of HCPs’ failures in recognition, diagnosis, treatment and reporting was developed based on literature review and expert opinion. A total of 360 HCPs were allocated to two groups. In one group their performance was tested by simulated patient (SP) methodology as gold standard, and one month later by the online tool to allow assessment of its sensitivity. In the other group, they were tested only by the online tool to allow assessment of any possible bias incurred by the exposure to SPs before the tool.

**Results:**

The sensitivity of the tool was (98.7%; CI 93.6–99.3). The overall agreement and kappa statistics were 96.6% and 85.6%, respectively. In the group tested by both methods, the failure proportion by SP was 86.1% (CI 80.1–90.8) and by tool 87.2% (CI 81.4–91.7). In the other group, the tool found 85.6% (CI 79.5–90.3) failures. There were no significant differences in detecting failures within or between the groups.

**Conclusion:**

The SMOT tool not only showed high validity for detecting HCPs’ failures in relation to CMSM, but it had high rates of agreement with the real-world situation, where malaria transmission has been interrupted. The tool can be used by program managers to evaluate HCPs’ performance and identify sub-groups, whose malaria vigilance should be strengthened. It could also contribute to the evidence base for certification of malaria elimination, and to strengthening prevention of re-establishment of malaria transmission.

## Background

The performance of health care providers (HCPs) in the management of suspected malaria is important for malaria control and elimination programs [1, 2]. Correct management of suspected malaria (CMSM) includes recognition, early diagnosis and prompt treatment with appropriate anti-malarial drugs aiming, as a minimum, to prevent severe disease and fatal outcomes [3, 4]. In elimination programs and after elimination has been achieved, correct case management should also include rapid notification, which may trigger a response to manage any transmission risk associated with the case [5].

Nevertheless, CMSM remains a significant shortcoming in many settings [6]. In particular, it is possible that in low transmission areas and countries progressing towards elimination, HCPs’ awareness may decrease due to the low incidence of malaria cases. Consequently, the absence of reported malaria cases may not necessarily mean that malaria is eliminated [1]. The alertness of general health services to malaria, known as *vigilance*, is a central component of malaria surveillance following elimination, and it must be established before malaria elimination certification can be granted [7]. The World Health Organization (WHO) malaria elimination guidelines include the indicator “percentage of patients with suspected malaria who received a parasitological test”, but provide no advice on measurement of the denominator [5].

The ideal method for assessing HCPs’ practice in relation to CMSM would be the observation of their performance when faced with real malaria patients. However, in countries close to elimination, these are so rare that such observation, while important, cannot provide a representative picture. Therefore, the next best assessment is observation of HCPs’ practice when faced with persons who *simulate* cases of suspected malaria. This methodology could be used in both low and high transmission settings but is likely to be costly on a large scale.

The study reported here aims to develop and validate a rapid survey tool called Simulated Malaria Online Tool (SMOT) for assessment of HCPs’ practice in relation to CMSM through comparison of HCP performance according to the tool with performance when faced with a person simulating suspected malaria. A validated rapid tool for assessing HCPs’ practice will be useful for assessing whether the current malaria surveillance system is able to detect, manage and report any new malaria case to the public health authorities; in other words, whether the surveillance system includes effective vigilance, which in combination with other measures can prevent the re-establishment of malaria transmission [7].

## Methods

### Study design and setting

The study was conducted in two phases during 2020–21 in a historically low malaria transmission area, East Azerbaijan Province of Iran, where no locally transmitted malaria case has been reported since 2005 [1]: Phase 1, developing a new tool, and Phase 2: implementation: evaluation of the tool in real life. The study population was HCPs to whom a febrile suspected malaria case might present in East Azerbaijan Province. Different groups of providers such as family physicians, physicians in hospital emergency rooms, private practitioners and specialists, and community health workers (CHWs) were included.

### Phase 1: SMOT tool development

#### Literature review

Several instruments/questionnaires have been developed to assess the management of malaria cases. However, due to the rarity of malaria cases in low transmission areas and/or countries in the elimination phase, there is a need for a tool that can simulate the real situation to determine what/how HCPs do when faced with a case of suspected malaria. Therefore, we carried out a systematic search through Medline, Web of Science, Scopus, and Embase up to 30 December 2020 to obtain relevant published records in English documenting the most relevant case history (description) of suspected malaria as well as clinical algorithms for suspected malaria recognition, diagnosis, and treatment.

The eligible studies included clinical algorithms, educational or practical malaria-related guidelines and reports, patient flow, signs and symptoms with high sensitivity and specificity in malaria case detection and diagnosis. The search results were used together with other inputs as described below to develop the contents of the tool to be compatible with suspected malaria.

#### Expert opinion involvement

The first draft of the SMOT tool was developed and reviewed by six experts with different expertise in malaria research, prevention, control and elimination at national (Iran and East Azerbaijan Province) and international levels (WHO). The members of the expert panel had experience in preparation of guidelines for malaria control and elimination. Records obtained from the literature review were combined with expert opinion for tool generation. The following criteria were considered for the tool development:

- It should be rapid, short, and easy to use.
- It should mimic a real suspected malaria case and include all steps and criteria in the management of suspected malaria: recognition, diagnosis, treatment, and notification.
- It must detect HCPs’ failures in suspected malaria case management with high sensitivity.
- It can be used through online, email, and virtual networks.
- It and its application should be inexpensive.

#### Health care providers’ feedback

The initial online version of the SMOT tool, in Persian, was shared among ten field-experienced experts, who provided comments for improving the tool. They included one infectious disease specialist, one epidemiologist, three family physicians (MD), two staff experts in the Department of Infectious Diseases (MSc and BS), two community health workers, and one health education specialist (PhD). All of them provided constructive feedback on the tool, including order of questions and scenarios, how to articulate questions to simulate SP methodology, to obtain HCPs performance in addition to awareness, clarification, and simplification of questions, and modification of scenarios. After the experts had filled out the tool, the following open questions were asked through face-to-face interview:

- Is the order of the questions or scenarios appropriate?
- Are the symptoms and descriptions mentioned in the questionnaire compatible with suspected malaria?
- Can the questionnaire assess the actual practice and situation when a suspected malaria case reports to a HCP?
- In your opinion, what changes should be made in the way of expressing and asking questions?

#### Content and application of the SMOT tool

The tool was designed to evaluate four practices in CMSM: (a) recognition of a suspected malaria case, (b) malaria testing and/or prescription of a correct malaria diagnostic test, (c) prescription of appropriate anti-malarial drugs, and (d) notification and/or submission of an urgent report of a confirmed malaria case.

**Fig. 1:**
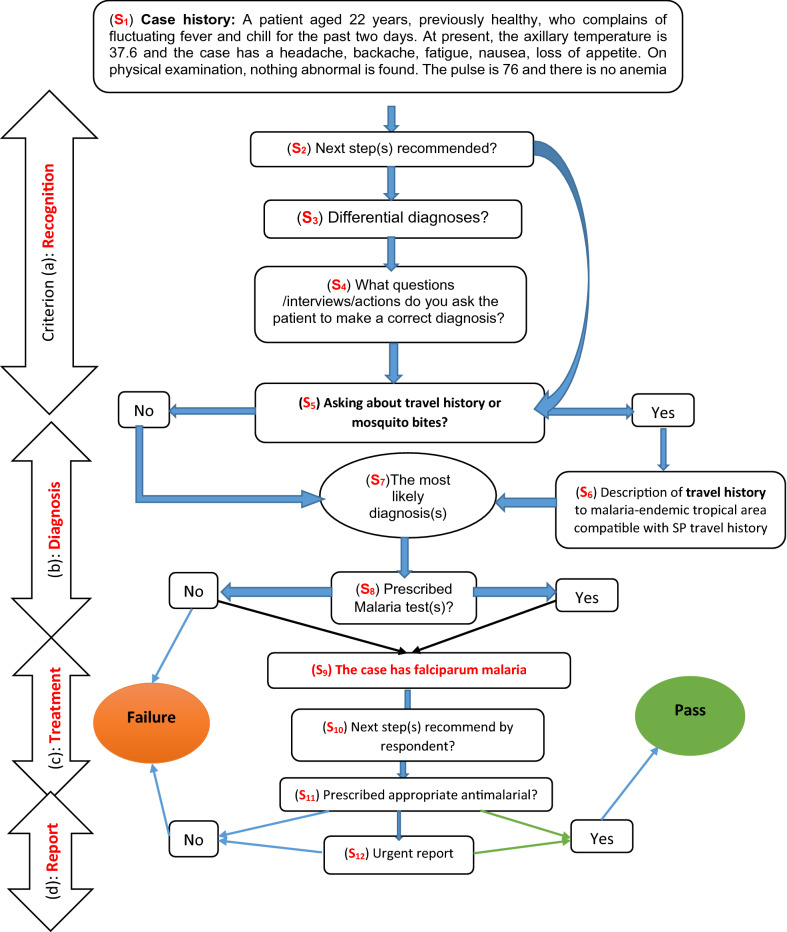
Flowchart of SMOT tool performance for detecting respondents’ failures in CMSM (S1–S12: Stage)

Figure 1 shows the SMOT flowchart. The tool mimics a febrile suspected malaria case presenting in an area, where there is no longer any malaria transmission, so that the case must have a history of travel to a malaria risk area. It is designed so that every question comes up based on the previous responses. For example, the details of the travel history are triggered if the respondent responds to the previous question(s) by asking about travel history. Answers to the tool questions and scenarios were open. There was no word limit for the responses to the SMOT questions. The details and sequence of the questions were as follows.

1. A patient aged 22 years, previously healthy, who complains of fluctuating fever and chills for the past two days. At present, the axillary temperature is 37.6 °C and the case has headache, backache, fatigue, nausea, and loss of appetite. On physical examination, nothing abnormal is found. The pulse is 76 and there is no anemia. Two PCR tests for Covid-19, one week apart, were negative, and a chest X-ray did not show any lesions. The results of para-clinical tests and evaluation of clinical symptoms rule out Covid-19 infection (see Fig. 1: S1).
2. What is your next step for this patient? (S2). If travel history was asked, the respondent was directed to S6 and given the following information: The patient has a travel history with a two-week stay on vacation or work in tropical Africa and returned to Iran through Dubai three weeks before the fever started.
3. If travel history was not asked, the respondent was asked: what is your differential diagnosis? (S3).
4. Subsequently, the respondent was told: To get the correct diagnosis, you may need more examinations or interview with the patient, or you may want to take several steps. What action or measures do you take to obtain the most likely diagnosis? Please indicate the exact term for the action or actions (S4). If the respondent now asked for travel history (S5), then s/he was directed to S6 (see above). If the respondent did not ask about travel history, s/he was directed to S7.
5. You may suspect several diseases. Based on the symptoms and history mentioned, and the test results, which disease(s) is/are your most likely diagnosis? (S7).
6. Which diagnostic test(s) do you prescribe based on your most likely diagnosis or diagnoses mentioned, in the previous question? (S8). (The aim of this question was to evaluate, if the provider prescribed malaria diagnostic test(s).
7. The case has malaria infection with laboratory-confirmation for falciparum malaria. What are your next step(s)? (S9). This information was provided, even if the respondent had failed in suspecting malaria and been classified as a failure on criterion (a).
8. What test(s) or exam(s) do you prescribe to diagnose malaria and its type of parasite? To which laboratory/center do you refer a patient with malaria for a diagnostic test? Within how long a period do you want to have the test results for this patient available to you? Please also specify the unit of duration (such as hour, day, or month …) (S10).
9. In your opinion, if there is a need to prescribe a drug, state the exact name of the drug or drugs, dose, and duration of its use. How will you provide anti-malarial drugs? (S11) (Note: If you are a non-physician, it is not necessary to answer these questions).
10. What other action or measures related to a confirmed malaria case do you think you need to take? (S12).

The tool link was sent through email and/or virtual networks. Respondents were aware that they were undergoing an assessment but not that it was about malaria. Whenever a participant had completed the tool, the investigator was alerted by email and provided the details of all the responses.

The data received was entered into the software. Responses were evaluated by one of the investigators (HA) who worked in the health system (unit for disease control/ malaria expert). The presence of outliers, bias or imprecision was assessed before data entry. The tool categorized respondents into two main groups (failure, and pass). Respondents were included in the “failure category” if their answers failed in at least one of the four criteria (a), (b), (c) and (d). As shown in Fig. 1 and explained above, downstream measures could be assessed, even if a respondent failed in the prior criteria.

#### Content validity

The final version of the SMOT tool was shared among 10 experts, who were asked to rate instrument items in terms of clarity and relevance to the underlying construct on a 4-point ordinal scale: (1) not relevant, (2) somewhat relevant, (3) quite relevant, (4) highly relevant. To obtain Content validity index (CVI), the number of experts who assigned a rating of 3 or 4 to the relevance of each item was divided by the total number of experts.

Regarding Content validity ratio (CVR), the experts were requested to specify whether an item is necessary for operating a construct in a set of items or not. To this end, they were asked to score each item from 1 to 3, *not necessary, useful but not essential,* and *essential*,‏ respectively. The content validity ratio is computed as CVR = (N_e_–N/2)/(N/2), in which the N_e_ is the number of panelists indicating “essential” and N is the total number of panelists. The numeric value of CVR was determined by Lawshe Table. CVR varies between –1  and 1. A high score indicates a higher degree of agreement on the necessity of an item. The necessity of the tool is confirmed if the overall score is above 0.7 [8, 9].

### Phase 2: Implementation; evaluating the tool by comparison with simulated patients

#### Definition and presentation of the Simulated Patient method as a gold standard

*Simulated Patient* (SP) technique was used as a gold standard to estimate the validity of the tool in comparison with SP (sensitivity of the tool in detecting HCPs' failures in CMSM, and detected failure proportions (p) by the tool and SP methodologies). Simulated Patients a.k.a. standardized patients, surrogate patients, or mystery patients are healthy persons trained for role-playing as a case of the condition of interest. SP methodology has been used as gold standard and in medical education in several studies [10–12]. The scenarios for SP methodology were generated based on our literature review and malaria expert inputs, tailored to assess HCPs' readiness and practice in low or no malaria transmission areas [1, 7]. As with the tool, the suspected case would have acquired the infection elsewhere.

#### Definitions of failures in the correct management of suspected malaria (CMSM)

A HCP encountering a suspected malaria case was considered a failure, if one or more of the following criteria were fulfilled [13]:

- Does not elicit a travel history and does not perform or request a diagnostic test for malaria. However, the exceptional case, where a provider has a patient tested without a travel history is accepted as a pass;
- Does not channel the patient with suspected malaria to have appropriate diagnostic testing performed within 24 h of the encounter;
- Does not ensure appropriate treatment within 12 h after a test result positive for malaria;
- Does not report the case to the appropriate public health authority within 12 h after a test result positive for malaria.

The failure definitions are almost identical to those applied with the SMOT, but the SP methodology did not allow for the possibility of telling the respondent that the patient had falciparum malaria if s/he had not had the patient tested. Thus, with the SP methodology, criteria (b), (c) and (d) could only be assessed for respondents, who passed on (a).

#### Personnel and role-playing for the SP methodology

The SP cases were trained university students (engineering, law, education, language, literature). The study excluded students of medicine or biology, as they could easily be drawn into a discussion about diagnostic options. The study included 10 SP persons since malaria experts and authors reached consensus that one SP person could role-play a suspected malaria case for 18 HCPs (in all 180 HCP participants).

HCPs were not informed of SP cases, and they provided care and treatment as usual for other patients. The details of the role-play and observations to be recorded by the SPs were as follows:

The SP case history was compatible with tool methodology and described the following signs and symptoms: fever going up and down for the last two days, but absent at presentation, accompanied by headache, backache, fatigue, nausea, loss of appetite, with no other symptoms. If a provider prescribed a Covid-19 test, a negative result was provided.

*Recognition* The provider must extract the travel history to make an accurate diagnosis. If the provider asked about the travel history, the SP responded travel history and two-week stay on vacation or work in tropical Africa and return to Iran through Dubai three weeks before the fever started. All questions and practices of the provider were observed and noted after the encounter(s). The SP had to pay attention to:

- Travel history: yes/no; if yes, for how long does it cover?
- Mention of malaria as diagnostic possibility: yes/no
- Referral for medical care? If yes, where?
- Consultation with specialists? Who at which institution?

Diagnosis: If malaria or any other diagnostic tests were prescribed by the provider, the following elements were observed by the SP:

- What kind of diagnostic test(s) were performed?
- Where was the test performed (name the institution/laboratory)?
- How many hours would it take before the test result were available from the time, when the test was ordered or the patient referred?

*Treatment* If malaria diagnostic test(s) had been prescribed, the SP was in 50% of cases equipped with a fake positive result issued by the most used reference laboratory in the area based on an arrangement made by the investigators with that laboratory. The SP viewed and audited the therapeutic practice of the provider, and the following elements were recorded after finishing the visit: anti-malarial prescriptions (dosage and type), consultation with specialists, time interval between malaria testing and the start of treatment, and hospitalization.

*Reporting* This could take place in the absence of the SP cases. Therefore, we checked at the healthcare institution that would normally receive the disease report, whether the SP has been reported. We ensured that the SP was not counted in the national malaria statistics.

#### Data collection (in SP methodology)

A 2-section paper form was applied. The first section was filled out through interview with SPs after every single visit by the SP volunteer to the HCP. The second was completed by monitoring the provider’s behaviour including timeliness for up to 48 h after the visit by the SP volunteer through checking the public health system (unit for disease control) to obtain any malaria notification and prescribed anti-malarial drugs through the SP volunteer’s insurance booklet and/or prescription to evaluate the correct malaria testing and treatment practices.

### Indicators to measure the validity of the tool in comparison with SP methodology in detecting HCPs failures in the correct management of suspected malaria

The study used four indicators to estimate the validity of the SMOT tool in comparison with SP methodology, as a gold standard, in detecting HCPs failures in the CMSM. Table 1 shows indicators definition and calculation in the current study in detail.

**Table 1:** Indicators for estimating the validity of SMOT tool in comparison with SP methodology in detecting healthcare providers’ failure in the correct management of suspected

No.	Indicator type		Definition in the current study
1	Sensitivity and specificity		Sensitivity was the ability of SMOT tool to correctly identify HCPs who failed in the CMSM; and specificity was the ability of SMOT tool to correctly identify HCPs who passed the CMSM
2	HCPs failure proportions (p)	Overall proportion (failed)	The number of HCPs recognized failed by the tool/SP methodologies in all four criteria (a-d) were divided into all tested HCPs
		By failure criteria (c, d)	The number HCPs recognized failed in sub-failures criteria (a, b, c, d) separately by the tool/SP methodologies were divided into all tested HCPs
3	Agreement (reliability)	Overall agreement (observed)	The proportion of True Positive (TP, real failed) plus True Negative (TN, real passed) divided by all values: (a + d)/(a + b + c + d) × 100) [14]
		Kappa statistic	K was measured inter-tool reliability in non-chance agreement of HCPs failures: $\kappa \equiv \frac{{p_{o} - p_{e} }}{{1 - p_{e} }}$ where *p*_*o*_ is the relative observed agreement between tool and SP, and *p*_*e*_ is the chance agreement. The K ranged 0 to 1. If the tool and SP are in complete agreement then k = 1 [15]
4	Likelihood ratio (LR)	LR^+^	To measure how much the odds of the failure increase when the tool is positive (HCP failure) in comparison with SP methodology, and calculated through $\frac{Sensitivity}{1-Specificity}$ [15]
		LR^−^	To measure how much the odds of the pass increase when the tool is negative (HCP pass) in comparison with SP methodology, and calculated through $\frac{1-Sesitivity}{Specificity}$[15]

### The study design and sampling to estimate the validity measures of the tool in comparison with SP

#### Health care provider sampling and allocation in two groups of A and B

**Fig. 2:**
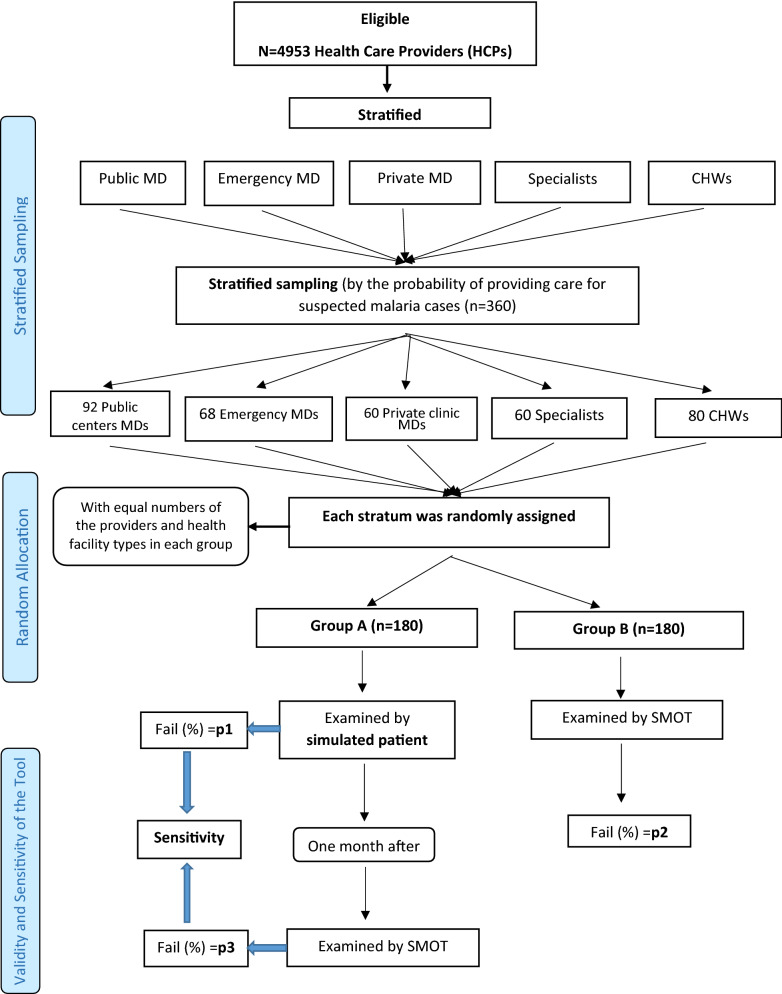
Healthcare provider’s inclusion, stratification sampling, and assessment of failure proportions in CMSM and assessment of validity and sensitivity of the tool. 1. Between groups A and B comparison of SP and tool (p1 with p2). 2. Within group (A) comparison of SP and tool; p1 with p3; (sensitivity of tool in comparison with SP). 3.Between group comparison of tool (with itself), p2 with p3; the difference incurred by performing tool one month after SP

HCPs were stratified by category and then randomly assigned to two groups, A and B, as described in detail below. Providers in group A were assessed by SP methodology and after one month, by the tool methodology. In group B, providers were assessed only by the tool methodology (Fig. 2).

In the first stage, all eligible HCPs in East Azerbaijan Province were stratified into five strata: general Medical Doctors (MDs) in public healthcare centers, MDs in hospital emergency rooms, MDs in private clinics, specialists, and CHWs [36]. The medical specialists were: Infectious, Internal, and Emergency Medicine.

The probability of providing care for suspected malaria cases in each stratum was determined based on expert opinion through a consensus meeting and examination of records of confirmed malaria cases in the study area. Then, 360 HCPs were randomly selected from the strata including 92 MDs in public health care centers, 68 in emergency rooms, 60 in the private clinics, 60 specialists, and 80 CHWs.

In the second stage, HCPs in each stratum were randomly assigned to two groups, A and B, with equal numbers from each stratum allocated to each group. Additional details for sampling and allocation are shown in Fig. 2.

#### Sample size

The sample size was estimated based on Sakpal’s study on sample size determination in randomized controlled design [16] by setting α = 0.05, β = 0.2, the failure proportions of the providers through SP and tool methodologies respectively p1 = 0.2, and p2 = 0.1; the sample size was 180 for each group.

### Validity measures of the tool in comparison with SP

#### Comparison 1 (of failure proportions of the tool and SP in two intendant groups of providers, A and B).

In group A, SP methodology was applied to determine the proportion, p1, of providers, who failed as defined above by criteria (a–d). In group B, tool methodology was applied without previous exposure to SP to determine the proportion, p2, of providers, who failed by the same definition. The comparison of p1 with p2 provided an assessment of the performance of the tool compared with the gold standard, SP in detecting HCPs failures in two independent groups (A and B) of HCPs.

#### Comparison 2 (sensitivity, specificity and agreement of the tool in comparison with SP, within group A)

The HCPs in group A, who had initially been examined by SP, p1, were examined by the tool one month later. The study estimated the proportion, p_3_, of providers, who failed in CMSM by the tool. Comparing the failures within group A, considering SP as the gold standard, the sensitivity, specificity, observed agreement and kappa statistics, and likelihood ratios of the tool for detection of failures in comparison with SP were estimated as is usually done for comparison of diagnostic tests by a contingency table. More details for measuring of these indicators was presented in Table 1.

**Table:** 

		SP	
		Pass	Fail
Tool	Pass	TN	FN
	Fail	FP	TP

Sensitivity is TP/(TP + FN) and specificity is TN/(TN + FP)

*TP* True positive; *TN* True negative; *FP* False positive; *FN* False negative

#### Comparison 3 (the comparison of p3, proportion of failures by tool in Group A, with p2, proportion of failures by tool in Group B

The comparison 3 allowed assessment of the bias (difference) incurred by performing the tool on providers one month after the encounter with an SP). If the level of this bias was not considerable, the assessment of sensitivity and specificity (Comparison 2) could be considered valid (Fig. 2).

#### Comparison 4 (of the SMOT tool and SP by failure criterion (a–d)

As explained above, downstream measures including criteria (b), (c), and (d) could be assessed through the tool, even if a respondent failed in the prior criteria since the tool provided a malaria positive test result for all respondents. However, in SP methodology, downstream measures were evaluated only for the providers who passed criterion (a). The evaluation was conducted by providing a fake positive test result in 50% of cases. As the denominators corresponded to the number of participants assessed for each criterion in the two methodologies, the failure proportions in all criteria (a–d) could be compared.

### Statistical analysis

SPSS software (version 19.0, Chicago, IL, USA) was used for data analysis. Student’s t-test was used for comparison of continuous variables, or Mann–Whitney test, when data distribution was not normal. Chi-square (*χ*^2^) test was used to compare failure proportions between groups. Fisher’s exact test was used to analyse failure proportions within group A, when at least one cell of a 2 × 2 table had an expected value below 5.

Agreement percentage and kappa statistic in detecting failure and pass cases were calculated between tool and SP methodologies. Sensitivity, specificity, and the likelihood ratio (LR) for failure results (LR +) were calculated to understand how much the odds of the failure increase when a test (tool) is positive (HCP failure) in comparison with SP methodology. The LR + and LR − were calculated as $\frac{Sensitivity}{1-Specificity}$ and $\frac{1-Sesitivity}{Specificity}$, respectively [34, 35].

## Results

### Literature review

A total of 447,845 records were identified by the search. Of those, 447,474 were removed due to duplication or based on title and abstract screening so that 371 studies were eligible to be assessed in full text. Finally, only10 records were selected for tool development based on their relevance to recognition of suspected malaria in areas with no or very low transmission. Four of these were published between 1990 and 1999, three between 2000 and 2009 and three between 2010 and 2019.

Findings of literature review indicated that patients with temperature ≥ 37.5 °C or history of fever, shivering, feeling hot, pallor or splenomegaly, vomiting, reduced food intake, and absence of rash and cough were common and reliable signs and symptoms with some sensitivity to predict malaria parasite infection (Table 2). However, most of the included studies suggested that clinical algorithms have little utility in malaria diagnosis (which must be highly sensitive due to the potential severity of the disease) and perform even worse in low transmission settings and older age groups.

**Table 2:** Key features and algorithms for febrile patients compatible with suspected malaria for developing case description and contents of SMOT tool and SP

First author-Ref	Year	Country	Clinical algorithm and key diagnostic criteria	Major Findings/recommendations
Muhe, [24]	1999	Ethiopia	Fever with a previous malaria attack or pallor or splenomegaly had sensitivities of 80% and 69% and specificities of 65% and 81% in high and low risk settings, respectively	Health workers should be trained to detect pallor and splenomegaly because these two signs improve the specificity for malaria
Ong Lean Suan, [25]	1990	Singapore	Integrated Malaria Expert system (IMEX): was implemented as four modules, initialization, diagnosis, treatment, and drug information. Decision-making (diagnosis) was based on input data and information	IMEX is a tool to assist medical personnel in the diagnosis and treatment of malaria, to improve management of malaria patients and to formalize and document knowledge on malaria
Thwing, [26]	2017	Senegal	A year-long study in 16 health posts was conducted to determine an algorithm’s capacity to identify patients with *Plasmodium falciparum* infection: ≥ 37.5 °C or history of fever; performed RDT, absence of cough, sputum, sore throat, skin rash, drainage in the previous 2 days	In all but the lowest malaria transmission zone, use of the algorithm excludes an unacceptably large proportion of patients with malaria from receiving an RDT at their first visit, denying them timely diagnosis and treatment
Weber, [27]	1996	Gambia	Fever above 38 °C and without intermittent fever and/or moderate to severe pallor, shaking was associated with the presence of parasitaemia Compared with the pediatrician’s diagnosis, the sensitivity and specificity of the Integrated Management of Childhood Illness (IMCI) algorithm was 87% and 8% for malaria parasitaemia (any level), and 100% and 9% for malaria parasitaemia (above 5000 parasites/µl)	The draft IMCI algorithm was not proven for malaria. Its use without microscopy would result in considerable overtreatment, especially in a low transmission area or during a low transmission season in countries with seasonal malaria
Duodu, [28]	2014	Ghana	An algorithm for malaria diagnosis and treatment using fuzzy logic was designed and simulated using MATLAB 7.8.0 via 13 malaria suspected cases; it included: *Imputed* data: age, sex, temperature, pulse rate, BMI; *Process*: Diagnosis; *Output*: prescribed drug. Prescribe drugs based on the degree of infection	The medical doctors’ diagnosis had accuracy of 61.5%, while the designed algorithm was 76.9% accurate
Mwangi, [29]	2005	Kenya	A clinical algorithm was generated based on examination of 1602 persons of all age groups for a range of signs and symptoms and malaria parasitemia.. problems were considered on the basis of their association with malaria. The age-optimized algorithms could identify about 66% of cases among those < 15 years of age but only 23% of cases among adults	Clinical algorithms therefore appear to have little utility in malaria diagnosis, performing even worse in the older age groups,
Bojang, [30]	2000	Gambian	Symptoms which contributed to the malaria: feels hot, absent of rash and cough, vomiting, reduced feeding, sleeping, and shivering 88% sensitivity and 62% specificity; compared with the pediatrician	The algorithm could be used for malaria diagnosis
Chandramohan, [31]	2001	India	Sign and symptoms of 1945 children and 2885 adults who presented with fever were recorded. Fever, shivering, chill, No cough and runny nose, feels hot to touch were common. Sensitivity: 60%, specificity: 61%)	malaria diagnosis in areas of low endemicity requires microscopy to be accurate
Case management, Guide for tutors, [32]	2012	WHO	This training module described many useful malaria suspected and confirmed case studies with various signs and symptoms and clinical features Common signs and symptoms used in the case histories included fever (in most cases was ≥ 37.5), vomiting, poor feel and nutrition, headache, backache, fatigue, nausea, absence of rash and neck stiffness Pulse rate, blood pressure, and travel history compatible with suspected malaria were reported in the most case studies	This training module on malaria case management has been developed to support the staff involved in malaria control and elimination programmes in the effective organization of malaria diagnosis and case management services
Redd, [33]	1996	Malawi	The rectal temperature of 37.7 °C or higher, splenomegaly, or nailbed pallor was 85% sensitive in identifying parasitaemic children and 41% specific	These results suggest that better clinical definitions are feasible, that splenomegaly and pallor are helpful in identifying children with malaria, and that much overtreatment of children without parasitaemia could be avoided

#### Content validity

Based on expert opinions, the overall necessity of the items of the tool was 97.0%. The overall appropriateness and clarity of the tool was obtained 96.4%, and 95.0%, respectively. The appropriateness and clarity of the tool was ≥ 80.0% for all items of the tool.

### Baseline characteristics of the HCPs

A total of 360 HCPs (180 in each group) were enrolled. In group A, HCPs were examined by the SMOT tool one month after SP methodology in order to make a paired comparison between SP and the tool. In group B, HCPs were evaluated by the SMOT tool. The distributions of demographic characteristics and of HCPs by professional category and health facility category were well balanced between the two groups and there were no significant differences (Table 3). The mean age was 38.8 ± 10.2 and 39.6 ± 10.3 in groups A and B, respectively. The proportion of females was 54.4%. MDs constituted the majority at 220 (61.1%) of HCPs.

**Table 3:** Demographic and baseline characteristics of the healthcare providers by the study groups

Variables	Healthcare providers (n = 360)		Total	P-value
	Group A (n = 180) (SP-tool)	Group B (n = 180) (tool)		
Age				
Mean ± SD	38.87 ± 10.2	39.66 ± 10.3	39.27 ± 10.2	0.471
Sex				
Female	(57.77) 104	(51.11) 92	196 (54.4)	0.244
Male	(42.22) 76	(48.88) 88	164 (45.6)	
Public community health center	(53.33) 96	96 (53.33)	192 (53.33)	0.958
Heath care facility category				
Emergency ward	34 (18.88)	34 (18.88)	68 (18.89)	
Private clinic/facility	(16.66) 30	(16.66) 30	60 (16.66)	
Public specialized clinic	(11.11) 20	(11.11) 20	40 (11.11)	
HCP category				
Medical Doctor	(61.11) 110	(61.11) 110	220 (61.11)	0.974
Specialist	(16.66) 30	(16.66) 30	60 (16.66)	
Community health worker	(22.222) 40	(22.22) 40	80 (44.44)	
Employment/position				
Public contract	(17.22) 31	(15.55) 28	59 (16.38)	0.422
Private contract	45(25)	(23.88) 43	88 (24.44)	
Official	(36.11) 65	54 (30)	119 (33.05)	
Commitment plan	(21.66) 39	(17.77) 32	71 (19.72)	
Work experience				
≤ 6 months	13 (7.22)	16 (8.88)	29 (8.05)	0.722
7–12 months	8 (4.44)	12 (0.66)	20 (5.55)	
1–2 year	31 (17.22)	32 (17.77)	63 (17.50)	
2–5 year	16 (8.88)	11 (6.11)	27 (7.50)	
5–10 year	29 (16.11)	34 (18.88)	63 (17.50)	
> 10 year	83 (46.11)	75 (41.66)	158 (43.88)	

#### Validity of the tool in comparison with SP methodology

In group A, HCPs were tested by the SP methodology as gold standard, and one month later by the SMOT tool to allow assessment of its sensitivity. The sensitivity of the tool was 98.7%; (95% CI 93.6–99.3) in comparison with SP methodology in detecting failures in CMSM, while the specificity was 84.0%; (95% CI 64.1–95.4) (Table 4). The overall agreement percentage and kappa statistics (non-chance) of the tool and SP methodologies were 96.6%, and 85.6%, respectively. Kappa statistics indicated that the SP and the SMOT tool have more than 85.0% agreement after removing chance agreements. The positive and negative likelihood ratios (LR + and LR −) were 6.17 and 0.015, respectively. LR + indicated how much the odds of the failure (true positive) increase when the SMOT tool is positive (HCP failure) in comparison with the SP methodology. While LR- indicated that in the true pass (true negative) results of the SMOT tool, what is the proportion of false negative (false pass) to true negative (true pass) in comparison with the SP methodology.

**Table 4:** Sensitivity and validity of the tool for detection of HCPs’ failures in the correct management of suspected malaria (in Group A as a within-group comparison)

	Tool	Simulated Patient (SP)		Total
		Pass	Fail	
Comparison of SP and tool methodologies in detecting HCP failures in CMSM	**Pass**	21	2	23
	**Fail**	4	153	157
	**Total**	25	155	180
Validity measures	Sensitivity	98.7%; (95% CI: 93.6 – 99.3)		
	Specificity	84.0%; (95% CI: 64.1 – 95.4)		
Agreement measures	Agreement percentage	96.6%		
	kappa	85.6%		
Likelihood ratio (LR)	LR^+^	6.17		
	LR^−^	0.015		

CMSM: Correct management of suspected malaria

CI: Confidence interval

### Comparison of the overall failure proportions between group (A and B)

*In group A*, the overall failure proportion of the providers was 155/180 (86.1%; CI 80.1–90.8) and 157/180 (87.2%; CI 81.4–91.7) by SP and tool, respectively (Table 5). Likewise, i*n group B*, the SMOT tool found the overall failure proportion of 154/180 (85.6%; CI 79.5–90.3). Between and within group analysis and comparison found no significant differences (overlapping CIs) in failure proportions of SP and tool methodologies (P = 0.535 for the between group comparison and P = 0.624 for the within-group A comparison). Furthermore, we found no significant difference (overlapping CIs, P = 0.688) in the failure proportions measured by the SMOT tool in group A and in group B, between-group comparison of the tool with itself, (see also Table 5 for more detail).

**Table 5:** Comparison of HCPs failures proportion and 95% CIs by tool and SP methodologies in the correct management of suspected malaria

Methodologies for assessment HCPs		Comparison of SP and tool (within and between groups) P-value	Comparison of tool with itself (between groups A and B) P-value
Group A (n = 180)			
SP before	p1: 155 (86.1%; 80.1–90.8)	p1 with p3: 0.624*	p2 with p: 0.688**
Tool after	p3: 157 (87.2%; 81.4–91.7)		
Group B (n = 180)			
Tool only	p2: 154 (85.6%; 79.5–90.3)	p1 with p2: 0.535**	

*HCPs* Healthcare providers; *p1, p2, and p3* failures Proportion; *p1 with p2* between groups A and B comparison of SP and tool; *p1 with p3* within group (A) comparison of SP and tool; (sensitivity of tool in comparison with SP); *p2 with p3* Between group comparison of tool (with itself); the difference incurred by performing tool one month after SP

^*^Fisher’s exact test

^**^Chi-square (*χ*^2^)

The highest sensitivity of the tool (100.0%) was observed among specialists, CHWs, and emergency wards. The failure proportions by subgroup ranged from 73.3% to 92.5% among different HCP categories; and from 73.3% to 94.1% among health facility categories. In group A, SP (before) vs tool (after), the highest HCP failure proportions (92.5% and 92.5%) were found in CHWs, while the lowest (80.0% and 76.6%) were found in specialists (Table 6). Likewise, in group B, the lowest failures (73.3%) were among specialists. Public specialized clinics seemed to have better performance than other health facilities. The study found no significant differences in failure proportions (p) between SP and tool methodologies nor between the various HCP or health facility categories (p > 0.05).

**Table 6:** HCPs’ failure number (percentage) in the correct management of suspected malaria by HCP and health facility category with comparison between groups

Failures in the sub-groups	Group A (n = 180)			Group B (n = 180)	P-value*
	*SP (before)*	*Tool (after)*	Sensitivity** (%)	*Tool*	
HCP type					
MD (n = 220)					
Failure	**94 (85.45)**	**97 (88.18)**	**95.87**	**100 (90.90)**	0.411
Pass	16 (14.54)	13 (11.81)		10 (9.09)	
Specialists (n = 60)					
Failure	**24 (80.00)**	**23 (76.66)**	**100.0**	**22 (73.33)**	0.510
Pass	6 (20.00)	7 (23.33)		8 (26.66)	
CHWs (n = 80)					
Failure	**37 (92.50)**	**37 (92.50)**	**100.0**	**32 (80.00)**	0.073
Pass	3 (7.50)	3 (7.50)		8 (20.00)	
Health facility type					
Public community health center (n = 192)					
Failure	**83 (86.45)**	**84 (87.50)**	**97.6**	**79 (82.30)**	0.664
Pass	13 (13.55)	10 (12.50)		17 (17.70)	
Emergency wards (n = 68)					
Failure	**30 (88.23)**	**30 (88.23)**	**100.0**	**32 (94.11)**	0.270
Pass	4 (11.77)	4 (11.77)		2 (5.90)	
Private or self-clinics (n = 60)					
Failure	**27 (90.00)**	**28 (93.33)**	**96.4**	**28 (93.33)**	0.694
Pass	3(10.00)	2 (6.77)		2 (6.77)	
Public specialized clinic (n = 40)					
Failure	**15 (75.00)**	**16 (80.00)**	**93.3**	**15 (75.00)**	0.642
Pass	5 (25.00)	4 (20.00)		5 (25.00)	

^*^Between-group comparison (SP in group A & Tool in group B) of failures by cadre and health facility types

^**^sensitivity of the tool in detecting HCPs failures (paired comparison between SP and the tool by sub-group)

### Comparison of failure proportions of the tool and SP by sub-failures criteria (a-d)

The performance of tool and SP methodologies in detecting failures was similar regarding failure proportions for all criteria (a–d). There were no significant differences in the proportions of failures on criteria (a–d) in the within and between groups comparisons of the SP and the SMOT tool methodologies (p > 0.05) (Table 7). Regarding failure proportions by sub-failure criteria (a–d), failure in criterion (a), recognition of suspected malaria, was the most common in both SP and tool methodologies (73% vs 73%), while the lowest proportions of failures were with criterion (b): 16.6% and 15.5% in SP and tool, respectively. The pass proportions in the study groups ranged between 12 and 15%, and there were no significant differences in between and within group comparisons.

**Table 7:** Number and (percentage) of failures in the correct management of suspected malaria by failure criterion (a–d) and study groups

Failure criterion*	Group A (n = 180)		P-value (comparison of SP and tool within group A)	Group B (n = 180)	P-value (comparison of SP and tool between group)
	SP (before)	Tool (after)		Tool	
(a) Not elicit travel history or suspicion of malaria	131 (72.77)	131 (72.77)	0.988	126 (70.00)	0.816
(b) Not/inappropriately tested for malaria	4 (16.66)	28 (15.55)	0.387	22 (12.22)	0.446
(c) Not prescribed appropriate anti-malarial treatment	13 (54.16)	101 (56.11)	0.633	98 (54.44)	0.820
(d) Lack of notification	11 (45.83)	93 (51.66)	0.452	89 (49.44)	0.833
No failures criteria (pass)	25 (13.88)	23 (12.77)	0.455	26 (14.44)	0.766

^*^For criteria b, c and d, the denominator with SP included only respondents, who passed (49) on criterion a, of those, 24 (50%) were evaluated by the fake positive results; while with the Tool, it included all respondents

Thus, the denominator for criteria (b) (c), and (d) with the SP column is 24

## Discussion

This is the first study known to us for developing and validating a survey tool to measure HCPs’ alertness and practice in relation to CMSM in an elimination setting. The study findings showed that the SMOT tool claims to have a very good agreement with the real conditions and it can be used instead of the real malaria case in settings where there is no malaria cases and/or it is rare, to evaluate HCPs' performance and malaria surveillance systems. Our tool was developed based on a literature review combined with expert opinion to be applicable online and correspond to an encounter with a patient with suspected malaria. It allows the detection of failures of HCPs in CMSM according to four criteria, briefly stated: a, recognition of suspected malaria, b, diagnostic testing, c, appropriate treatment after a positive test and d, reporting a confirmed case.

Content validity, appropriateness, clarity, and necessity of the tool items were assessed by standard methods, and all found to be above 95.0%; according to expert and HCPs’ opinions, the tool content was sufficient.

To estimate the sensitivity and specificity of the tool in detecting HCPs’ failures, the study compared results of the tool methodology with results obtained by a standardized SP methodology applied one month earlier to the same sample of HCPs. The comparison indicated a sensitivity of almost 99% and specificity of 84%.

The overall agreement of the tool in comparison with SP was more than 96%. Application of the kappa statistic showed that the actual (non-chance) agreement of the tool was as high as 85% [17]. The LR^+^ and LR^−^ were 6.17 and 0.015, respectively, which indicates high confidence for both types of likelihood [18].

A comparison with application of the tool only to another random sample of HCPs from the same universe, who had not been exposed to SPs, indicated that the exposure to SP before the tool had not led to any bias: The tool showed a failure rate of 87.2% when preceded by the SP method against 85.6% in the sample with no application of SP. Therefore, in other settings, to assess the tool methodology with SP as gold standard, a paired comparison with SP in a single sample of HCPs can be considered. This will require smaller a sample size than the comparison of two independent samples. However, the apparent absence of an effect of exposure to a patient presenting a positive malaria test was surprising, so this result, which could perhaps have resulted from the overwhelming attention to Covid-19, when the study was carried out, should be interpreted with caution. While the study population had been selected to be representative of all relevant HCPs in East Azerbaijan Province, it must be recognized that their performance was rather uniform, with more than 70% failures. It is possible that in an HCP population with more varying results, as could be expected in an area with higher malaria risk, the correspondence in results obtained by the two methods might not be so strong.

Regarding failures on criteria (a–d), the majority (72.7%) were observed for criterion (a) (recognizing suspected malaria). This suggests that malaria has disappeared from the minds of most providers after the local elimination of the disease around 2005 [1]. Still, once suspected malaria is recognized, the next steps of case management were often done correctly. Therefore, providing regular simple warnings and education through the health care system prioritizing alertness to the possibility of malaria could probably significantly improve the management of suspected malaria. The tool could be particularly useful, when applied at different points in time after such education to evaluate the effect and the need for refreshment.

The highest failure proportion was found among CHWs (92.5%), while specialists had the lowest, but still high, proportion (73.3%). Although these differences did not reach statistical significance, the trends were as expected, thus supporting the validity of the methods.

Other malaria programmes and research institutions might be interested in carrying out similar studies with variations in the questionnaire tool and/or the SP method. With local adaptation, the tool may be adopted in elimination programmes. It may be used to indicate geographic areas or HCP categories or health facilities, where there is a particularly severe need for intervention. The data generated by this tool can also be useful in relation to malaria certification by WHO. An adaptation of our tool could also be considered in high transmission settings, but it is not certain that the study findings can be extrapolated to areas, where HCPs see malaria cases almost daily. A rapid online tool based on similar principles might prove useful and cost-effective, but its development should probably be based on a local identification of the priority problems in case management [19].

The adoption of our methodology in a program should be supplemented with the definition by a competent committee of a threshold value for to the maximum acceptable proportion of providers who do not manage cases of suspected malaria adequately. An additional threshold value could indicate a lower proportion of failures at which a given area must be prioritized for intervention. With such thresholds defined, it should be easy to set up Lot Quality Assurance Sampling (LQAS) [20–22] for large-scale application of the tool.

## Strengths and limitations

The main strength was using malaria and health care experts for developing tool contents and constructs and conducting stratified random sampling and random assignment of the providers in two groups to control the between group error and bias in detecting HCPs’ failures.

SP methodology was considered the only option for a gold standard to assess the tool’s validity in an area, where indigenous and imported malaria is rare. It proved to be easy to train volunteers to simulate the role of suspected malaria. Thanks to the good-will of laboratories, fake parasite positive results could be provided in 50% of SP cases for monitoring HCPs practice after being exposed to confirmed malaria cases.

Unfortunately, the proportion of HCPs failing in CMSM was high, from 73.3% in medical specialists to 92.5% in CHWs. The fact that a travel history had to be actively elicited by the HCPs may have caused overestimation of the proportion of HCPs who would fail in CMSM in real life. Another reason for the high failure rates could be that the study was carried out during the covid-19 pandemic, when HCPs’ attention was to a large extent fixated on this one disease [23]. However, we provided/informed providers regarding COVID-19 negative test results in two methodologies.

## Conclusions

The SMOT tool showed high validity and sensitivity for detecting HCPs’ failures and their practice in CMSM in a setting, where malaria transmission has been interrupted. The study found that there were no significant differences in the validity of SP and tool methodologies in detecting HCPs' failures. Therefore, the SMOT tool can be used instead of a real malaria case in settings where malaria transmission is interrupted or malaria cases are rare to evaluate the HCPs’ practice and malaria surveillance systems in the CMSM.

While the tool and the SP methodology are liable to underestimate the performance that would likely be observed, if HCPs were faced with true malaria patients, the SMOT tool provides a relevant, sensitive, objective and efficient method for evaluating HCPs’ practice and the vigilance function of the surveillance system. It can easily be adapted to be applied also in settings, where malaria is close to elimination, but still transmitted at low level.

## Data Availability

The datasets generated and/or analyzed during the current study are available from the corresponding author on reasonable request.

## Abbreviations

HCPs: Healthcare providers
CMSM: Correct management of suspected malaria
SMOT: Simulated Malaria Online Tool
MD: Medical doctor
CHWs: Community health workers
SP: Simulated patient
LQAS: Lot quality assurance sampling
